# Structural dynamics in chromatin unraveling by pioneer transcription factors

**DOI:** 10.1007/s12551-024-01205-6

**Published:** 2024-07-04

**Authors:** Andrea Orsetti, Daphne van Oosten, Roxana-Geanina Vasarhelyi, Theodor-Marian Dănescu, Jan Huertas, Hugo van Ingen, Vlad Cojocaru

**Affiliations:** 1 Bijvoet Center for Biomolecular Research, Utrecht University, Utrecht, Netherlands; 2 Faculty of Biology and Geology, Babeş-Bolyai University, Cluj-Napoca, Romania; 3 Faculty of Chemistry and Chemical Engineering, Babeş-Bolyai University, Cluj-Napoca, Romania; 4 STAR-UBB Institute, Babeş-Bolyai University, Cluj-Napoca, Romania; 5 Yusuf Hamied Department of Chemistry, University of Cambridge, Cambridge, England; 6 Max Planck Institute for Molecular Biomedicine, Münster, Germany

**Keywords:** Structural dynamics, Chromatin, Pioneer transcription factors

## Abstract

Pioneer transcription factors are proteins with a dual function. First, they regulate transcription by binding to nucleosome-free DNA regulatory elements. Second, they bind to DNA while wrapped around histone proteins in the chromatin and mediate chromatin opening. The molecular mechanisms that connect the two functions are yet to be discovered. In recent years, pioneer factors received increased attention mainly because of their crucial role in promoting cell fate transitions that could be used for regenerative therapies. For example, the three factors required to induce pluripotency in somatic cells, Oct4, Sox2, and Klf4 were classified as pioneer factors and studied extensively. With this increased attention, several structures of complexes between pioneer factors and chromatin structural units (nucleosomes) have been resolved experimentally. Furthermore, experimental and computational approaches have been designed to study two unresolved, key scientific questions: First, do pioneer factors induce directly local opening of nucleosomes and chromatin fibers upon binding? And second, how do the unstructured tails of the histones impact the structural dynamics involved in such conformational transitions? Here we review the current knowledge about transcription factor–induced nucleosome dynamics and the role of the histone tails in this process. We discuss what is needed to bridge the gap between the static views obtained from the experimental structures and the key structural dynamic events in chromatin opening. Finally, we propose that integrating nuclear magnetic resonance spectroscopy with molecular dynamics simulations is a powerful approach to studying pioneer factor–mediated dynamics of nucleosomes and perhaps small chromatin fibers using native DNA sequences.

## Introduction

Cells only need to express a fraction of their genetic information, which depends on cell type, differentiation status, and external stimuli. Key in the regulation of gene expression are transcription factors (TFs), proteins that bind to short regulatory DNA sequences to either activate or silence the transcription of genes (Maston et al. 2006; Spitz and Furlong 2012; Lambert et al. 2018; Kim and Wysocka 2023). However, the DNA in eukaryotes is not generally accessible for TF binding, as a large part of it is packed in nucleosomes, the basic unit of chromatin.

**Fig. 1:**
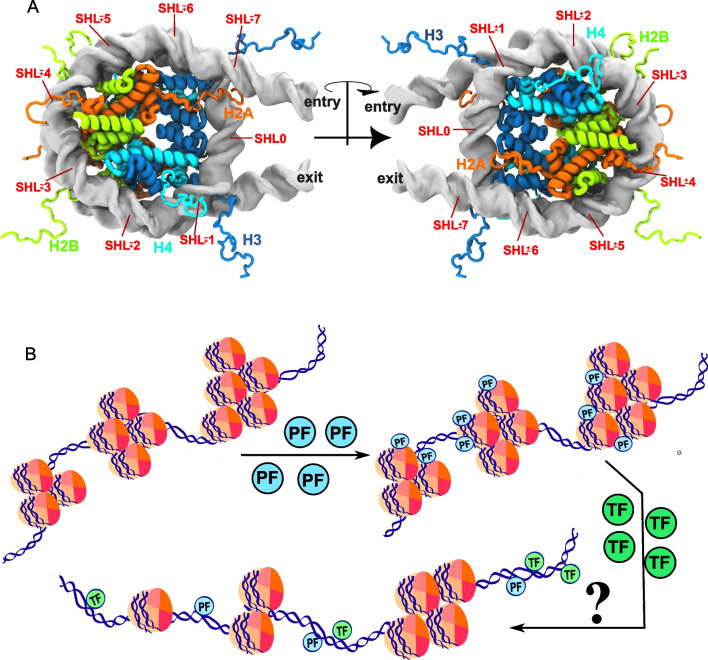
Pioneer factors open chromatin and operate at the nucleosome level. **A** Structure of a 168-bp nucleosome with the Widom DNA sequence with the four different core histones, including tails (Huertas et al. 2021), shown in different colors (color code in the figure) and the position of each superhelical location (SHL) indicated. **B** Schematic model of pioneer factor (PF) activity. Nucleosome binding is likely relevant in the early stages, when the PF binds target DNA sites in closed chromatin, and may contribute to chromatin opening. Chromatin opening and recruitment of cooperating non-pioneer transcription factors (TF), together with chromatin remodelers and histone modifiers, are thought to drive activation of gene expression. The question mark (?) indicates that the structural features and dynamics involved in PF function at different levels are still not understood

The wrapping of DNA around the histone octamer core, into nearly two superhelical turns with two closely packed DNA gyres (Luger et al. 1997), drastically reduces the exposure of the DNA major and minor grooves for TF binding (Michael and Thomä 2021) (Fig. 1A). Alterations in major and minor groove width induced by the curvature of DNA in the nucleosome (Richmond and Davey 2003; Chua et al. 2012) may further reduce TF binding. In addition, binding of linker histone H1 (Zhou et al. 2015; Bednar et al. 2017; Dombrowski et al. 2022) and inter-nucleosome interactions may further impede access of TFs to the DNA, both in highly compacted chromatin fibers (Schalch et al. 2005; Song et al. 2014; Adhireksan et al. 2020) and in native chromatin fibers that are more loosely compacted (Ou et al. 2017; Jentink et al. 2023; Hou et al. 2023).

Nucleosomes are considered to impose a general barrier to transcription (Knezetic and Luse 1986; Kornberg and Lorch 2020). This is highlighted in the selective chromatin packaging of enhancers, regulatory DNA elements that control the expression of distal, tissue-specific genes (Field and Adelman 2020). Enhancer TF binding sites are not chromatinized in cells where the gene is expressed, permitting binding of tissue-specific TFs, but packed in chromatin in other cell types preventing binding of TFs (Tillo et al. 2010; Barozzi et al. 2014).

The conundrum of how cells can develop and change their transcriptional program may be explained in part by the pioneer factor model (Zaret and Carroll 2011). In this model, a special class of TFs called pioneer transcription factors (PFs) can bind their DNA target motifs in closed chromatin and initiate opening of chromatin, ultimately resulting in the activation of silenced genes (Zaret 2020; Larson et al. 2021; Balsalobre and Drouin 2022; Bulyk et al. 2023) (Fig. 1B). Due to this pioneering activity, PFs are considered master regulators of cell fate and identity. This is not only relevant in differentiation, but also in cellular reprogramming. Here, the genetic program of differentiated, somatic cells is rewired to create an induced pluripotent state (Takahashi and Yamanaka 2006), involving large-scale changes in chromatin accessibility (Knaupp et al. 2017; Li et al. 2017).

**Fig. 2:**
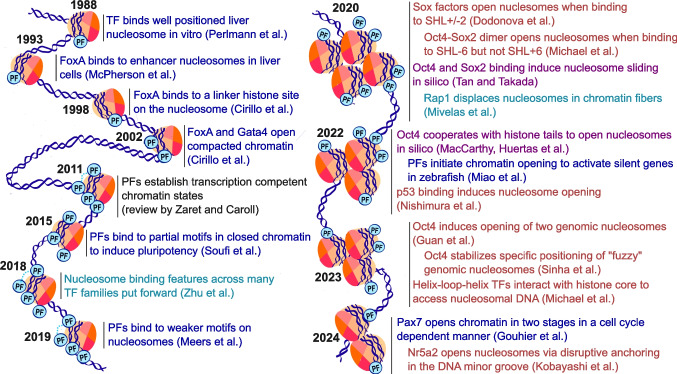
Timeline of selected landmark studies on pioneer factors. The color indicates the type of study: blue—molecular/cell biology; black—review; cyan—biochemistry/biophysics, red—structural biology; purple—molecular dynamics simulations. The number of nucleosomes per year reflects the number of selected papers. The number of PFs bound to each nucleosome reflects the number of PFs studied in the corresponding article. Four PFs connected by the light blue dashed curve stand for multiple PFs. We would like to emphasize that his selection is biased by our personal perspective and restrained by the limited space. We acknowledge that the understanding of PF action is advanced by many more crucial studies that were not included here, for which we apologize to all authors of those studies

A canonical example PF is FoxA, a TF which is required for activation of the albumin gene (Gualdi et al. 1996; Lee et al. 2005). The Zaret lab found that FoxA, whose DNA binding domain (DBD) is similar to that of the linker histone, binds a positioned nucleosome in the albumin enhancer (McPherson et al. 1993; Cirillo 1998) and can cause the opening of chromatin structure by displacing linker histone H1 (Cirillo et al. 2002). Since these first discoveries, a series of cellular, biochemical, and structural studies have shed light on the molecular mechanism of PFs (Fig. 2 shows a timeline of selected landmark studies). Mechanistically, PFs work together with other transcription factors (Gualdi et al. 1996; Soufi et al. 2012, 2015; Chronis et al. 2017; Mayran et al. 2019), chromatin remodelers (King and Klose 2017; Frederick et al. 2023), and histone modifiers (Gouhier et al. 2024) to reorganize chromatin structure and convert enhancers from a silenced to an activated state, capable of stimulating transcription.

The key initial event is the recruitment of the PF to closed chromatin. As such, PFs will have several layers of chromatin accessibility (Mansisidor and Risca 2022) to navigate: they have to access the inactive heterochromatin compartment, scan the compacted chromatin, and ultimately bind their target site, even if initially embedded in a nucleosome. Some PFs such as FoxA stably bind nucleosomes in the activated state of the enhancer (see also below). However, this may not be true for all PFs. For example, Pax7 (a pioneer factor in pituitary gland development) causes nucleosome eviction at targeted sites (Gouhier et al. 2024). Still, PFs will encounter nucleosomes in the early stages of pioneering chromatin for ultimate gene activation. Also, the sites pioneered by Pax7 are enriched in nucleosomes initially (Mayran et al. 2018).

Recently, several high-resolution structures of PFs bound to nucleosomes have been resolved (Fig. 2), which have been discussed in several excellent recent reviews (Michael and Thomä 2021; Morgunova and Taipale 2021; Luzete-Monteiro and Zaret 2022). The emerging view is that nucleosome binding typically involves the recognition of a partial DNA recognition motif. In some cases PF binding induces significant DNA distortions, nucleosome repositioning (nucleosome sliding), or partial unwrapping of the nucleosomal DNA (nucleosome opening) (see below), which could be crucial in initiating chromatin opening and facilitating access for other factors. Here, we focus on the initial interaction of PFs with chromatin and review several key aspects of PF-mediated chromatin opening. First, we examine the cellular and biochemical evidence for PF binding to chromatin and nucleosomes. We compare the degree of nucleosome opening in recent PF–nucleosome structures, highlighting the role of histone tails, DNA sequence, and PF binding site location. Finally, we propose how an integrated approach based on nuclear magnetic resonance spectroscopy (NMR) and molecular dynamics (MD) simulations may be exquisitely suited to unveil the dynamical aspects of the PF–nucleosome interaction in initiating chromatin opening.

## Pioneer transcription factor activity from genome-wide experiments

**Fig. 3:**
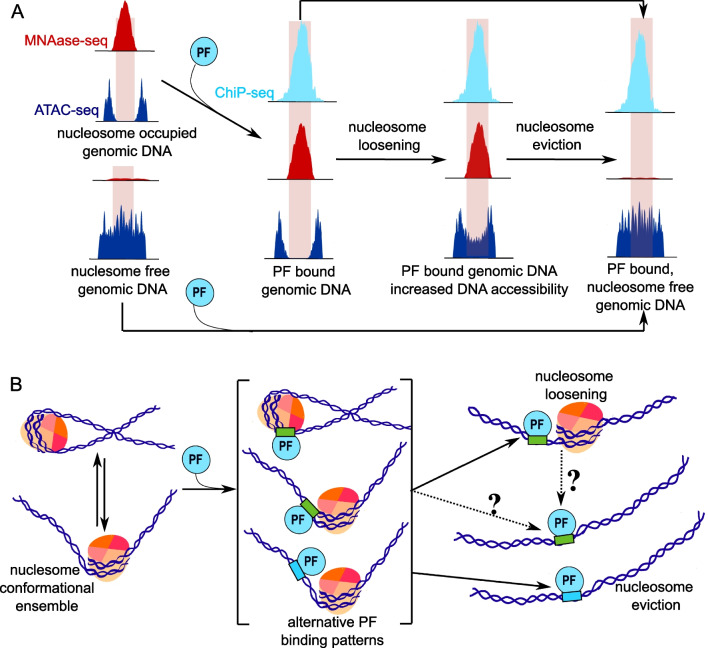
Outcomes of PF binding to chromatin. **A** Perspective from cell population genomic data. Chromatin footprinting identifies genomic locations occupied by nucleosomes (MNase-seq) and accessible regions (ATAC-seq). Comparison against PF binding sites from ChIP-seq is used to identify nucleosome binding by PFs. Binding may result in nucleosome loosening or even nucleosome eviction. **B** Perspective from structural data. Transient exposure of DNA due to chromatin and nucleosome dynamics (left), and DNA target motif strength influence binding pattern of PF to the nucleosome (middle), with “weak” (in green) motifs favoring nucleosome binding and “strong” (in cyan) motifs favoring free DNA binding. This induces or stabilizes nucleosome opening, modifying the accessibility of the recognized DNA motifs. Finally, further opening of the nucleosome may lead to nucleosome loosening or even nucleosome eviction (right). The question marks indicate that the mechanisms involved in those steps are yet to be discovered. For simplicity, we do not show any additional proteins that are recruited upon PF binding at different steps depicted in the figure

To unambiguously demonstrate the pioneer activity of a TF, one would ideally be able to follow a particular TF molecule, detect its binding to a target DNA motif inside a nucleosome, and monitor the impact on local chromatin accessibility, the recruitment of other (non-pioneer) TFs, chromatin remodelers, and co-activators and ultimately the activation of gene expression, all within the context of the cell. Clearly, current technologies only allow to observe specific aspects of this scenario, typically averaged over a bulk of cells. Identification of pioneer activity in cells thus relies on the comparison of experimentally determined TF binding sites with chromatin footprinting results and gene expression changes, obtained in parallel experiments (see Fig. 3A).

Identification of functional pioneer activity, or functional PF–nucleosome interactions, is, however, not without some caveats. Since eukaryotic TFs recognize short motifs of 6–12 bp, it is inevitable that TFs show widespread non-functional binding (Wunderlich and Mirny 2009). Also, it can be challenging to link a binding site to an enhancer element of a specific gene, due to the distal arrangement of enhancers and genes and enhancer redundancy (Field and Adelman 2020; Kim and Wysocka 2023). Finally, since TF and chromatin mapping experiments are typically performed on a bulk of cells and in independent experiments, there is at least the theoretical possibility that the correlation between a PF binding site and a positioned nucleosome may not occur in the same cell at the same time.

### Mapping nucleosome positions, pioneer factor binding sites, and chromatin accessibility

A map of experimental nucleosome positions can be obtained via micrococcal nuclease digestion followed by sequencing (MNase-seq) (Schones et al. 2008). Here, the DNA restriction enzyme MNase is used to fully digest free DNA regions, resulting in the isolation of the DNA sequences embedded in nucleosomes. With proper care to prevent over-digestion of DNA in which the MNase also digests the nucleosome (Mieczkowski et al. 2016; Chereji, Bryson, and Henikoff 2019), MNase-seq provides a direct readout of nucleosome occupancy and positioning along the genome (Fig. 3A).

The genomic binding sites of TFs are typically mapped using chromatin immunoprecipitation followed by sequencing (ChIP-Seq) (Johnson et al. 2007; Park 2009). In ChIP-Seq, TF binding sites are determined by sequencing DNA fragments that are obtained after purification of cross-linked protein-DNA complexes using a specific antibody to the protein of interest (Fig. 3A).

Chromatin accessibility is most often determined using an assay for transposase-accessible chromatin followed by sequencing (ATAC-seq) (Buenrostro et al. 2013; Grandi et al. 2022). In ATAC-seq, a hyperactive Tn5 transposase fragments accessible DNA and directly tags the fragments for sequencing. While ATAC-seq can be used to map nucleosome positions (Schep et al. 2015), ATAC-seq is commonly used to map highly accessible regions in the genome (Fig. 3A). These accessible regions are usually interpreted as nucleosome-free regions. Importantly, this assumption is not necessarily true: depending on reaction conditions and the local accessibility to MNase (15 kDa) and Tn5 (100 kDa), nucleosomes can be identified in high chromatin accessibility regions from ATAC-seq (Roberts et al. 2021).

The methods described above allow us to infer the nucleosome binding of a PF from the correlation between mapped nucleosome positions and PF binding sites in independent experiments. By fusing the MNase to protein A, which binds strongly to a conserved region in many antibodies, the MNase can be directed to cleave at sites surrounding an antibody-bound protein without cross-linking. This approach is known as Cleavage Under Targets and Release Using Nuclease (CUT&RUN) (Skene and Henikoff 2017). CUT&RUN can essentially combine ChIP-Seq and MNase-seq in a single experiment, providing a direct read-out of PF-nucleosome binding (Meers et al. 2019).

### FoxA: evidence for pioneering activity from ChIP-seq and MNase-seq

The identification of pioneer activity can be illustrated using a study on the FoxA PF in mouse liver (Iwafuchi-Doi et al. 2016). Through ChIP-Seq experiments, a genome-wide map of approximately 30,000 FoxA binding sites could be obtained at near-bp resolution. This map of FoxA binding sites was compared against experimental nucleosome positions from MNase-seq. Nucleosome binding was substantiated by a sequential ChIP experiment in which FoxA-bound nucleosomal fragments were shown to contain core histones.

Further experiments showed that FoxA bound most frequently the dyad region of the nucleosome and that FoxA deletion resulted in the increased presence of H1 and reduced presence of cooperating liver non-pioneer TFs at liver-specific FoxA-targeted enhancers. Ultimately, approximately 100 FoxA binding sites could be linked to liver-specific enhancers containing a nucleosome whose accessibility was FoxA-dependent and where FoxA deletion resulted in downregulation of the (predicted) related gene. Thus, pioneer activity of FoxA is required to maintain an open state of the enhancer and depends on the ability of FoxA to bind nucleosomes and displace linker histone H1, as in the “nucleosome loosening” scenario of Fig. 3A.

### Oct4 and Sox2: pioneering activity from integrating ChIP-seq, MNase-seq, and ATAC-seq

Similar findings have been reported about the maintenance of pluripotency in embryonic stem cells (ESCs) by pioneer factors, and master regulators of pluripotency, Oct4 and Sox2 (Teif et al. 2012). An Oct4 mutant protein that showed free DNA binding but no nucleosome binding in vitro caused a reduction of chromatin accessibility in ATAC-seq at sites where wild-type Oct4 colocalized with a positioned nucleosome, thus strongly indicating a crucial role for nucleosome binding of Oct4 in maintaining open chromatin at these sites (Roberts et al. 2021). In the case of Sox2, ATAC-seq studies showed that acute depletion of Sox2 in ESCs resulted in rapid re-establishment of closed chromatin at a subset of Sox2 sites. This implies that the pioneer activity of Sox2 is required to maintain accessibility at these sites, including the enhancer for pluripotency gene *Klf2* (Maresca et al. 2023).

Soufi and co-workers uncovered a critical role for nucleosome binding by Oct4, Sox2, and Klf4, (together “OSK”), in the reprogramming of fibroblasts to iPSCs. In the early stage of reprogramming, OSK are bound mainly to closed chromatin (Soufi et al. 2012), at sites that are enriched for nucleosomes in the pre-existing chromatin as determined from MNase-seq data in pre-infected fibroblasts (Soufi et al. 2015). Indeed, OSK can bind a reconstituted nucleosome from one of the identified sites, the enhancer of *Lin28B*, a gene involved in pluripotency (Soufi et al. 2015). Moreover, successful reprogramming was found to be dependent on nucleosome binding. An Oct4 mutant with selectively impaired nucleosome binding was not able to generate iPSCs (Roberts et al. 2021).

### Pax7: time-course ATAC-seq identifies a role for cell division in pioneering activity

An exciting recent study integrated a series of ChIP-seq and ATAC-seq experiments to dissect the time course of pioneer activity of Pax7 during cellular reprogramming of corticotrope cells from the pituitary gland into melanotrope cells (Gouhier et al. 2024). It was found that Pax7 targets silenced enhancers in closed, facultative heterochromatin and initiates loss of linker histone and a change from repressive to active histone modifications. Interestingly, full activation of and chromatin opening at the enhancer required cell division.

The authors propose that cell division allows repartitioning of the poised enhancer from the heterochromatin into the active chromatin compartment, where chromatin remodelers and histone modifiers subsequently establish a fully active enhancer where nucleosomes are evicted (“nucleosome eviction” scenario in Fig. 3A). To what extent Pax7 engagement to chromatin involves nucleosome binding has not been explicitly tested, although earlier work from the Drouin lab indicated that initial chromatin interaction may involve nucleosome binding (Mayran et al. 2018).

### Impact of motif density and degeneracy on PF-nucleosome binding modes

To get detailed insight into DNA vs. nucleosome binding the CUT&RUN method was used to map the binding profile of 10 TFs, including PFs FoxA, Oct4, and Sox2, during the differentiation of ESCs to the endoderm stage. Notably, all PFs showed both binding to both free and nucleosomal DNA. For FoxA, nucleosome binding was relatively strongest at the onset of differentiation, in line with the pioneer model. Strikingly, analysis of the DNA sequences at the free vs. nucleosomal DNA-binding sites indicated that strong, optimal recognition motifs are enriched in the free DNA sites while nucleosomal binding sites have in majority suboptimal, weak motifs in which there are a number of mismatches to the consensus motif. The authors propose that at sites containing strong recognition motifs, PFs can efficiently trap transiently exposed DNA, due to intrinsic nucleosome dynamics (Li and Widom 2004; Fierz and Poirier 2019) and/or exposure during replication, while at weak affinity sites, PFs will bind the nucleosomal DNA (Meers et al. 2019) (see Fig. 3B).

Similar targeting of weak, or partial, motifs that are compatible with nucleosome binding had also been observed in the initial chromatin engagement of OSK during reprogramming (Soufi et al. 2015). Because of the large number of such nucleosomal binding sites, it is likely that these represent a “scanning” mode of the PF, which raises the question of how the functional sites are selected. Recent work by Gibson et al. (Gibson et al. 2024) on the *Drosophila* PFs Zelda and Grainy suggests that chromatin opening at pioneered sites is related to prolonged PF occupancy encoded by a higher count of stronger, canonical recognition motifs compared to non-functional PF binding sites.

Motif density was also suggested to be a critical factor in discriminating PFs from non-PFs in a study of pioneer activity of FoxA and non-PF HNF4 during the programming of embryonic fibroblasts to induced endoderm (Hansen et al. 2022). When co-expressed, both FoxA and HNF4 engage chromatin at liver-specific enhancers, resulting in increased chromatin accessibility. Surprisingly, expression of non-PF HNF4 alone was sufficient to cause chromatin opening nearby liver-specific genes, albeit that pioneered sites by HNF4 had a higher motif count compared to those pioneered by FoxA.

### Nucleosome binding of pioneer and non-pioneer transcription factors

In conclusion, advanced cellular studies are unveiling increasingly more about the molecular mechanisms of PF function, while at the same time highlighting the tremendous complexity of gene regulation. In some cases, like FoxA, nucleosome binding seems integral to pioneer function, from initial chromatin binding to the final activated stage. In other cases, nucleosome binding may be restricted to the initial stage. Together with the observation that the DNA binding domains (DBDs) of many TFs can bind nucleosomes (Zhu et al. 2018) (see also below), it is clear that there is no rigid dichotomy between PFs and non-pioneer TFs: both can bind free and nucleosomal DNA. Still, as PFs have the unique, functional ability to activate target enhancers in closed chromatin, PFs need to have a special ability to navigate the presence of nucleosomes, either by binding or by displacing them. What factors determine the extent of nucleosome binding and the degree of stable complex formation remain largely unresolved.

## Location of transcription factor binding sites on nucleosomes

One key question for the elucidation of the mechanisms involved in the PF-nucleosome interaction is whether PFs bind preferentially to binding sites situated at specific locations on the nucleosome. The positions on the nucleosome are usually described by a parameter known as the superhelical location (SHL). By convention, every helical turn (~ 10 bp) of the nucleosome is assigned an SHL, with SHL 0 at the dyad and SHL ± 7 at the DNA entry and exit sites (or SHL − 7 and SHL + 7 at the 5′ and 3′ ends of genomic nucleosomes) (Fig. 1A) (McGinty and Tan 2021). It is plausible that the affinity of a PF to a certain DNA motif varies depending on the SHL where the motif is located because of differences in histone-DNA interactions, packing of the DNA gyres, and position of histone tails.

Moreover, the impact of PF binding on nucleosome structure and dynamics may differ depending on the recognized SHL (Fig. 3F). To map the locations of TF binding to the nucleosome, biochemical experiments such as DNA mutations or footprinting or more sophisticated single molecule biophysical assays such as DNA unzipping (Rudnizky et al. 2018) are typically required. From footprinting experiments, Perlmann and Wrange found in 1988 that the glucocorticoid receptor TF could bind its DNA target site in a reconstituted nucleosome, providing the first direct evidence for TF-nucleosome binding (Perlmann and Wrange 1988).

### Biochemical mapping of nucleosome binding locations for a large pool of transcription factors

Perhaps the most comprehensive study to date to systematically analyze the positional preference of roughly 30% of all transcription factors was performed by Zhu et al. (2018). They developed nucleosome consecutive affinity purification–systematic evolution of ligands by exponential enrichment (NCAP-SELEX) experiments. To determine the TF binding specificities and positions on the nucleosomes, they screened libraries of 147- or 200-bp DNA for sequences that assemble into reconstituted nucleosomes and are bound by TFs. Then, they recovered the bound DNA from the purified complexes by PCR and analyzed the enriched sequences after multiple selection rounds. By comparing screens with reconstituted nucleosomes and free DNA, they first confirmed that the nucleosome is a barrier for TF binding and can impact the binding orientation of many TFs.

Most interestingly, they provided a comprehensive map with the preferred binding positions on the nucleosomes for all TF analyzed. The map revealed that most TFs prefer to bind at the entry and exit sites on the nucleosomes. However, some TFs such as the members of the Sox family prefer to bind to more central SHLs (SHL0, ± 2). While some TFs bind both DNA gyres, other TFs bind to nucleosomes with a periodicity or totally asymmetric. Some interesting differences were observed among members of the same family. For example, while FoxA2 clearly prefers its DNA binding site at the entry site, for some other Fox factors such as FoxK1, FoxK2, FoxO1, or FoxE1, multiple locations including more central ones are possible. Some Fox factors (FoxO1 and FoxE1) also have a periodic preference pattern.

Zhu et al. (2018) also analyzed the effect of TF binding on nucleosome stability using an additional step in which they separated the nucleosome-bound and dissociated DNA in the last cycle of their NCAP-SELEX experiment. They showed that most but not all TFs destabilized the nucleosome. Some families of TFs (in particular ETS) show a position-dependent effect on nucleosome stability. Again they discovered some interesting differences between members of the same family. For example, while Pax7 has a clear destabilizing effect, Pax1 and Pax2 appear to not affect nucleosome stability.

### Identifying binding locations of individual pioneer factors on engineered nucleosomes

Other studies to map nucleosome binding positions have been carried out for several PFs using techniques such as electrophoretic mobility shift assays and single molecule fluorescence microscopy. In these, the binding of PFs to nucleosomes with motifs inserted at different locations into strong positioning nucleosome sequences was investigated (often Widom 601 (Lowary and Widom 1998)). For example, it has been found that Oct4 prefers to bind at the nucleosome ends (SHL ± 5/6) (Michael et al. 2020; Echigoya et al. 2020). In the case of Sox2, the outcomes of different studies have been somewhat contradictory. While the NCAP-SELEX experiment found Sox factors preferred binding to the nucleosome dyad or dyad-adjacent regions (also supported by Li et al. 2019), others have found that Sox2 has a preference for the nucleosome ends (Michael et al. 2020; Malaga Gadea and Nikolova 2023).

Interestingly, the cooperativity between Sox2 and Oct4 is the sole most important interaction for the induction of highly competent pluripotent stem cells (MacCarthy et al. 2024), but few studies examined this in the context of nucleosome binding. Several reports showed that Oct4 and Sox2 can form stable ternary complexes with the nucleosome only at the ends (Michael et al. 2020; Malaga Gadea and Nikolova 2023). By comparing a native, genomic with an artificial DNA sequence, it was found that Oct4 is able to increase the affinity of Sox2 for the nucleosome ends in the artificial nucleosome, but not in the genomic Lin28B nucleosome, possibly due to the orientation of the binding sites (Li et al. 2019).

Other PFs for which the nucleosome binding preferences were mapped include, for example, p53. From in vitro assays, it was found that p53 prefers nucleosome ends. This was supported by genomic evidence from lung fibroblasts (Yu and Buck 2019). In agreement with the NCAP-SELEX study, the bHLH transcription factors MYC-MAX and CLOCK-BMAL1 both exhibited a preference for the nucleosome ends (Michael et al. 2023). Notably, while GATA3 prefers nucleosome end binding in vitro, a genomic assay revealed that GATA3 motifs are enriched at SHL + 2 in productively remodeled loci, but GATA3 binds to the nucleosome ends at non-productive loci (Tanaka et al. 2020)*.*

### Identifying binding locations of individual pioneer factors on native nucleosomes

The major problem with all experiments using reconstituted nucleosomes is that the DNA sequences used are engineered sequences for which the nucleosome positioning is rather fixed, and the locations of the binding sites can be clearly mapped. However, native nucleosomes in cells are more dynamic, and it is far more difficult to unambiguously assign the location of the binding sites to precise SHLs. Native nucleosomes breathe, wrap and unwrap, or slide making a systematic mapping of TF binding preferences very challenging. Thus far, there are few examples in which binding-site locations have been identified on native nucleosomes selected from genome-wide studies. A recent preprint analyzing the binding of Oct4, Sox2, Klf4, and Myc (OSKM) to nucleosomes reconstituted using genomic target nucleosomes as observed in the study of Soufi et al. (2012) found that Sox2 and Klf4 bind the nucleosome center in a subset of targets, while inserting of the binding motif in a strong-positioning DNA sequence blocked binding (Tsompana et al. 2022).

Other studies analyzed in detail the Lin28B nucleosome, which is bound by all three OSK factors and selected from the initial study by Soufi et al. (2015). The location of the Oct4 and Sox2 binding site was proposed from footprinting experiments (Soufi et al. 2015; Roberts et al. 2021) and DNA mutations (Echigoya et al. 2020; MacCarthy et al. 2022). Interestingly, in the case of Oct4, DNA mutations identified an additional binding site on Lin28B compared to the sites originally found from ChIP-Seq and footprinting experiments. This site was located at SHL − 7 according to an initial assignment of nucleosome positioning based on MNase data (Echigoya et al. 2020; MacCarthy et al. 2022). This location was further confirmed by cryo-EM structures of the Lin28B nucleosome and by a recent structure of the Oct4-Lin28B complex. Interestingly, this last structure revealed an Oct4-mediated nucleosome sliding upon binding to this site which is shifted to SHL − 8 (Sinha et al. 2023), further highlighting the difficulty of precisely assigning binding site locations on native nucleosomes.

## Pioneer-factor induced nucleosome dynamics from experimental structures

To unambiguously identify the binding locations and geometries of PFs on nucleosomes, it is of utmost importance to resolve atomic resolution structures of PF-nucleosome complexes. This is necessary also for understanding the mechanisms of PF-mediated cell fate transitions and for potentially using the pioneering functions of TFs to optimize or manipulate such transitions. The structures of PF-nucleosome complexes resolved in recent years provide the first insights into PF-induced nucleosome opening. However, the structural link between nucleosome opening and chromatin opening is still missing (Fig. 3B).

### Large amplitude nucleosome opening in structures with Sox factors bound

**Fig. 4:**
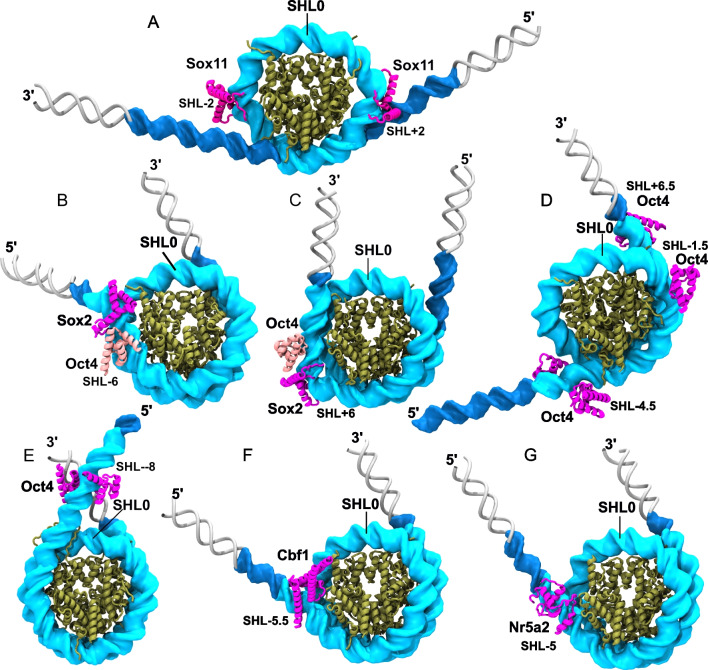
Nucleosome conformations in structures of pioneer factor-nucleosome complexes. **A** Sox11 bound at SHL ± 2 (PDB 6t7c). **B**, **C** Sox2-Oct4 dimer bound at SHL − 6 (PDB 6t90) (**B**) and + 6 (PDB 6yov) (**C**). **D** Oct4 bound at multiple sites to the Lin28B nucleosome (PDB 7u0g). **E** Oct4 bound to the SHL − 7 (SHL − 8 after nucleosome sliding) (PDB 8g8g). **F** Cbf1 bound at SHL − 5.5 (PDB 7ssa). **G** Nr5a2 bound at SHL − 5 (PDB 8pki). The histone core is in tan and the DNA visible in the structures is in light blue. In dark blue is the DNA construct used in the experiment and in grey are B-DNA segments modeled in to highlight the amplitude of the conformational opening

Because of difficulties in reconstituting PF-nucleosome complexes in vitro, it took roughly 20 years after the structure of a nucleosome core particle was determined (Luger et al. 1997) until the first structures of PF-nucleosome complexes were reported. Dodonova and co-workers from the Cramer lab reported the cryo-EM structures of Sox2 and its close homologue Sox11 bound to nucleosomes. They used a DNA sequence selected from the NCAP-SELEX experiments (Dodonova et al. 2020). Remarkably, 25 base pairs of DNA at each end of the nucleosome were not observed in the cryoEM map, suggesting that the nucleosome is in an open conformation with flexible ends (Fig. 4A). In general, Sox factors bind to the minor groove and induce a sharp bend in the DNA. The binding orientation and the Sox-induced deformation of nucleosomal DNA structure at SHL + 2 or SHL − 2 were different. Although the structures lack most of the histone tails, it was observed that a small fragment of the H4 tail is repositioned by Sox2/Sox11 binding at SHL + 2 (Dodonova et al. 2020). These structures provided the first atomic resolution capture of a PF-induced or stabilized open conformation of a nucleosome. They also revealed SHL-dependent structural features of PF-nucleosome complexes and histone tail repositioning upon PF-nucleosome binding.

### Alternative nucleosome conformations in structures with the Oct4-Sox2 heterodimer bound

Almost at the same time, structures of the ternary complex between Oct4, Sox2, and the nucleosome with the PFs bound to composite motifs inserted into the Widom 601 sequences were reported. At SHL − 6, Sox2 distorts the DNA and cooperates with Oct4 to partly release the DNA from the histone core (Fig. 4B). Interestingly, at SHL + 6, the motif is inverted, and while there is still strong local DNA distortion due to Sox2, the nucleosome is not open (Michael et al. 2020) (Fig. 4C). These structures were first to reveal another important aspect in nucleosome recognition by PFs, namely how PFs with multiple DNA binding domains interact with the nucleosome. Oct4 has two DNA binding domains (POU_S_ and POU_HD_) linked through a flexible linker. The POU_S_ is bound next to Sox2 in both structures with a protein–protein interaction interface similar to that on free DNA with a canonical Oct-Sox motif (Jerabek et al. 2014; Merino et al. 2015). The POU_HD_ was not observed in either structure, and it was proposed that the POU_S_ is sufficient for chromatin opening. Notably, later computational studies validated with experimental data challenged the idea that the POU_HD_ is not important for nucleosome recognition (Tan and Takada 2020; MacCarthy et al. 2022).

### Nucleosome opening and sliding in structures with Oct4 bound

More recently, the first cryo-EM structures of Oct4 alone bound to nucleosomes with genomic DNA sequences reported a range of binding modes and Oct4-induced nucleosome conformations. Guan et al. (2023) observed the binding of multiple Oct4 molecules to three different motifs on a Lin28B nucleosome (SHL − 4.5, SHL − 1.5, SHL + 6.5) (Fig. 4D). At SHL − 4.5, both the POU_S_ and POU_HD_ were bound and induced the unwrapping of 25 base pairs of DNA with the POU_HD_ wedging between the DNA gyres. Upon POU_HD_ deletion, the unwrapping was significantly impaired, clearly establishing the essential role of the POU_HD_. This mechanism resembles that reported by MacCarthy et al. (2022) from MD simulations of Oct4 bound to a different nucleosome at SHL + 5.5 (see below). At SHL − 1.5 and SHL + 6, only the POU_S_ was bound with only subtle effects on the nucleosome conformation (Guan et al. 2023). Interestingly, the positioning of the Lin28B nucleosome was similar to that reported in the absence of Oct4 in contrast to the report by Sinha et al. (2023) who proposed a mechanism involving an Oct4-mediated repositioning of the Lin28B nucleosome. In this study, Oct4 was bound to nucleosomes with the Lin28B and nMATN1 sequences with both domains on opposite sides of the DNA, mimicking the binding to free DNA (Fig. 4E). Remarkably, Sinha et al. (2023) proposed that the Lin28B nucleosome is a fuzzy nucleosome and that Oct4 traps the histone octamer in a position with the binding site originally located at SHL − 7 sliding to SHL − 8 and fully accessible. This binding site was previously proposed as the highest affinity binding site for Oct4 alone on Lin28B (Echigoya et al. 2020; MacCarthy et al. 2022). Moreover, Sinha et al. also reported that Oct4 affects the conformational ensemble of the H4 tail and that H3K27ac increases the cooperativity of Oct4 binding to different sites on the nucleosome. The latter claim was recently challenged (Lian et al. 2023), highlighting the scientific excitement around the topic of PF-nucleosome recognition.

### Diverse nucleosome conformations in structures with other pioneer factors bound

Whereas most structures of PF-nucleosome interactions determined to date are for Oct4 and Sox2, recently solved structures of other PFs bound to the nucleosomes have provided additional insights into the diversity of PF binding modes and structural effects on nucleosomes. For example, the binding of GATA3 with two of its Zn finger domains to SHL ± 5.5/6.5 on the Widom 601 nucleosome did not alter significantly the nucleosome structure (Tanaka et al. 2020). The guardian of the genome, p53, binds as a tetramer to the end of the Widom 601 nucleosome, opening the nucleosome (Nishimura et al. 2022). Moreover, its C-terminal domain contains an additional DNA binding domain that mediates non-specific binding and facilitates binding to the nucleosome dyad.

The MYC-MAX and CLOCK-BMAL1 dimers, members of the bHLH TF family, bind to nucleosomes at different sites to partially accessible binding sites, displacing nucleosomal DNA (Fig. 4F). Interestingly, these dimers also interact with the histone core, and their binding is enhanced by Oct4 binding (Michael et al. 2023). Finally, NR5A2, a key regulator of zygotic genome activation (Gassler et al. 2022) unwraps the nucleosomal DNA by a mechanism involving minor groove anchoring and DNA structure distortion (Kobayashi et al. 2024) (Fig. 4G). The NR5A2-induced unwrapping was confirmed using fluorescence resonance energy transfer experiments, highlighting the power of biophysical approaches to study PF-induced nucleosome dynamics (Kobayashi et al. 2024).

Taken together, these structures provide some important insights into the mechanisms of PF-nucleosome recognition. Unwrapping of up to 25 base pairs at DNA ends seems to be the most common mechanism by which PFs impact on nucleosome dynamics. However, the details of this unwrapping and how it affects chromatin fibers remain to be discovered. For example, it is not clear to what extent the local DNA distortion induced by some PFs is important for the unwrapping. And perhaps most importantly, it remains unclear how the histone tails influence the PF-mediated nucleosome unwrapping and chromatin opening. Although in some structures repositioning of histone tail fragments has been observed, none of the structures reveals the complete histone tails.

## Pioneer factor–induced nucleosome dynamics from molecular simulations

Given the limitations discussed above, the diversity of PF-nucleosome binding modes and PF-induced nucleosome dynamics, it is unlikely that the complexity of the mechanisms by which PF initiates the opening of chromatin can be understood from structure determination alone. An enormous number of structures would be required to decode the effect of DNA sequence, binding site location, histone tail dynamics, and posttranslational modifications. Therefore, using alternative approaches is crucial. In recent years, molecular dynamics (MD) simulations have provided an accurate description of biomolecular interactions and dynamics on continuously increasing time scales (Sanbonmatsu 2019). Such simulations can be performed at full atomistic resolution or with reduced, coarse-grained representation of the biomolecular systems.

### Sox2-induced nucleosome sliding from coarse-grained simulations

The first simulations of the Oct4 and Sox2 interactions with nucleosomes were performed using a coarse-grained representation (Tan and Takada 2020). Tan and Takada characterized spontaneous binding of Sox2 and Oct4 to the nucleosome which would be impossible to study with atomistic simulations. They found that Sox2 binding preference depends on the positional rotation of its binding motif, which Oct4 binding is more diverse, both its POU_S_ and POU_HD_ domains recognizing different partial motifs. Interestingly, they observed a preference for the binding of the POU_HD_ to the SHL − 7, a binding site which was later validated by experiments (Echigoya et al. 2020; MacCarthy et al. 2022). And most interestingly, they proposed a Sox2-induced sliding of the Lin28B nucleosome. Although to date Sox2-induced nucleosome sliding has not been observed in other studies, an Oct4-induced sliding has been proposed (Sinha et al. 2023).

### Nucleosome dynamics from atomistic simulations

While coarse-grained simulations may provide insights into larger-scale motions as those discussed above, they are missing the detailed physico-chemical nature of the atomic interactions. On the other hand, atomistic simulations are not suitable to study spontaneous binding, but they can reveal all the detailed structural dynamics involved in the interactions. Free nucleosomes have been studied extensively with atomistic MD simulations up to timescales of 15 µs (Shaytan et al. 2016; Huertas and Cojocaru 2021; Armeev et al. 2021; Peng et al. 2021; Li et al. 2022). Reviewed by Huertas and Cojocaru (2021), the main advantage of MD simulations is that nucleosome models could be simulated using virtually any sequence, provided there is accurate data on nucleosome positioning to build accurate starting models. Moreover, complete and/or modified histone tails can be included in these simulations. There are two major challenges for atomistic nucleosome simulations. One is the long time scales of nucleosome dynamics which make it hard to observe large-scale transitions on the time scales accessible in the simulations. Another related challenge is the simulation of the binding and unbinding of histone tails to/from different DNA segments within the nucleosome. Usually, histone tails collapse on one DNA segment and remain bound throughout a typical microsecond-long simulation (Shaytan et al. 2016; Armeev et al. 2021). However, large-scale motions of histone tails are needed for nucleosome opening and thus essential to simulate the effect of PF binding to nucleosomes (Huertas and Cojocaru 2021; MacCarthy et al. 2022). In a recent development, Peng et al. (2021) demonstrated that with a careful choice of parameters focusing on the water model, such transitions can be observed in longer simulations.

### Oct4 mediated nucleosome opening from atomistic simulations

**Fig. 5:**
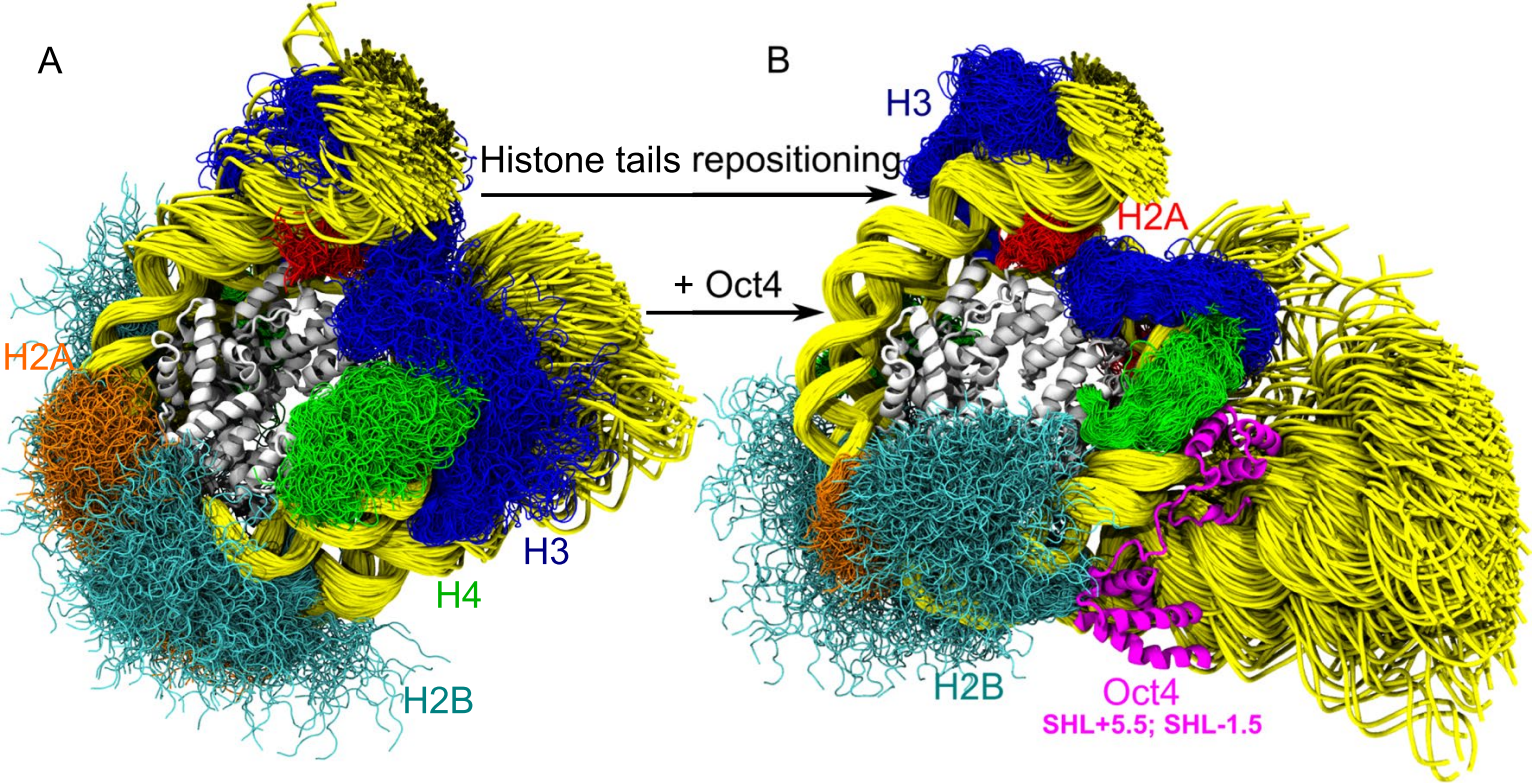
Dynamics of one Oct4-nucleosome complex from molecular dynamics simulations. (A) Three microseconds total time of MD simulations (3*1 µs per simulation) of the free Esrrb nucleosome (Huertas et al. 2021). (B) Three-microsecond-long MD simulation of the Oct4-Esrrb complex in which the nucleosome opened (MacCarthy et al. 2022). Snapshots at 20 ns time intervals are superimposed to highlight the dynamic nature of the histone tails (color code on the Figure) and the DNA (yellow). The core histones (white) and Oct4 (magenta) are shown only at the end of the simulations for clarity. One Oct4 domain (POU_S_) is bound sequence specifically to SHL + 5.5 while the second domain (POU_HD_) moves to bind unspecifically to SHL − 1.5 during the simulation stabilizing the open conformation of the nucleosome

MacCarthy et al. (2022) proposed a mechanism for the Oct4-induced opening of genomic nucleosomes from MD simulations (Fig. 5) even before structures of Oct4 alone bound to these nucleosomes were determined from experiments. After they experimentally validated all Oct4 binding sites using DNA mutations, they proposed that Oct4 stabilizes a partially open conformation of the Lin28B nucleosome when bound to SHL − 7 and induces a large opening of another nucleosome (from the Esrrb enhancer) when bound to SHL + 5.5. They revealed that both domains of Oct4 are important for nucleosome recognition and opening, a finding in agreement with experimental data. While one of the domains was bound to its sequence-specific site, the other one scanned the DNA non-specifically and stabilized open nucleosome conformations by wedging between the two DNA gyres.

### The role of histone tails in Oct4-mediated nucleosome opening

Perhaps the most important finding was that the position and conformational dynamics of H3 and H2A tails were essential for nucleosome opening both in the absence and presence of the PF and that the PF enhances this motion. In particular, the opening of the Esrrb nucleosome was only observed when the H3 tail was repositioned on the DNA around the dyad and all its contacts with the linker DNA were lost. They also observed that the pattern of interactions between the H4 tail and the DNA is dependent on Oct4 binding. These findings suggest that the repositioning of histone tails is required for PF-induced nucleosome opening. PF binding may stabilize specific nucleosome conformations with histone tail positions that facilitate binding site accessibility. Alternatively, PF binding may displace histone tails and induce their repositioning. However, this latter motion is almost impossible to sample in current state-of-the-art MD simulations.

### Comparison of molecular simulations with recent experimental structures

Many concepts proposed by MacCarthy et al. such as the Oct4-induced nucleosome opening, the roles of the two Oct4 DNA binding domains and the redistribution of the histone tail positions have also been confirmed in more recent experimental studies (Sinha et al. 2023; Guan et al. 2023). However, there are some important differences that highlight some of the challenges in MD simulations. Although the simulations did reveal that the POU_HD_ was bound sequence-specifically to SHL − 7 on Lin28B, they did not reveal nucleosome sliding as observed by Sinha et al. (2023). The positioning of the simulated Lin28B nucleosome was more similar to that observed in the structures of the free Lin28B nucleosome (Echigoya et al. 2020; Roberts et al. 2021). Furthermore, MacCarthy et al. did not observe the opening of Lin28B nucleosome upon binding of Oct4 at SHL − 4.5 as reported by Guan et al. (2023). Guan et al. also reported a structure with an Oct4-induced opening of the Esrrb nucleosome similar in nature to that observed in the simulations. However, the major opening observed in the cryo-EM structure occurred at the other end of the nucleosome (5′) and was triggered by the binding of two Oct4 molecules to different binding sites. Although one POU_S_ domain is bound on the site at SHL + 5.5 as in the simulations, the 3′ end of the nucleosome is closed in the cryoEM structure. The opening at the 3′ end may be less favorable than in the simulations because the 5′ end is already largely open, and the nucleosomes tend to open asymmetrically (Huertas and Cojocaru 2021; Armeev et al. 2021).

Most recently, MD simulations also revealed the dynamics of nucleosome opening by GATA3 and the differences in the dynamics upon binding of two different Zn finger domains of GATA3 (Ishida et al. 2023). Furthermore, Ozden et al. used a combination of molecular modeling and (all-atom) MD simulations to characterize the binding of Sox11 to the nucleosome at different positions (Ozden et al. 2023). They found that at SHL ± 2, Sox11 distorts the nucleosomal DNA (“shape reading”) and forms base-specific contacts with the DNA (“base reading”). However, at SHL ± 4 or the dyad (SHL 0), the shape reading is reduced, and the base-specific contacts are not observed. These observations were confirmed with hydroxyl radical footprinting and UV-laser footprinting experiments.

These studies show that MD simulations will play a pivotal role in future studies for understanding the mechanisms of PF-nucleosome interactions. In particular, they will be instrumental in studying the role of histone tails, binding site location, and DNA sequence in PF-mediated nucleosome opening.

## Pioneer factor and nucleosome dynamics from NMR spectroscopy

As emphasized above, one of the main challenges in MD simulations is the accurate sampling of the histone tail conformations in the context of the nucleosome. Therefore, it will be essential to combine MD simulations with experimental techniques in which biomolecular dynamics can be measured. NMR spectroscopy is a powerful tool to study conformational dynamics at the atomic scale due to the intrinsic sensitivity of the NMR signal to molecular motions. Spurred by technological developments in both solution and solid-state NMR, there have been an increasing number of NMR studies on the nucleosome over the last decade, highlighting conformational dynamics in the nucleosome and nucleosome-binding proteins (see for reviews (van Emmerik and van Ingen 2019; Musselman and Kutateladze 2022)).

### NMR reveals structures and dynamics in nucleosome-protein interactions

So far, only a few NMR studies focused on PFs and their interaction with nucleosomes. Fortuitously, the first high-resolution NMR study on the nucleosome focused on the interaction with the protein HMGN2 (Kato et al. 2011), an intrinsically disordered protein that, while not a PF, can decompact chromatin (Vestner, Bustin, and Gruss 1998), displace linker histone H1 (Catez et al. 2002), and is linked to active, cell type-specific enhancers (He et al. 2018). The protein was found to anchor to both core histones and nucleosomal DNA, thus positioning its chromatin-unfolding domain near the histone H3 tail and linker histone binding site (Kato et al. 2011).

Another early work demonstrated that the binding mode of a histone tail and DNA binding protein could be resolved (van Nuland et al. 2013). Most importantly, NMR studies have revealed insights into several structural and dynamical aspects of nucleosomes and nucleosome-protein interactions that are relevant for PFs as well: the dynamics and interactions of the histone tails (Zhou et al. 2012; Gao et al. 2013; Jennings et al. 2023; Sun et al. 2023), dynamics in the histone octamer (Kitevski-LeBlanc et al. 2018; Shi et al. 2018, 2020, 2023), the impact of protein binding on histone core dynamics (Sinha et al. 2017; Sanulli et al. 2019), the impact of histone modifications on nucleosome dynamics (Shoaib et al. 2021; Kim et al. 2023; Nosella et al. 2024) molecular interactions in phase-separated chromatin (Elathram et al. 2023), and the impact of higher-order structure on the histone-DNA interface (Smrt et al. 2023). While usually focused on the protein signal, recent developments are opening the possibility to also probe the structure and dynamics of the nucleosomal DNA (Abramov et al. 2020; Conroy et al. 2022; van Emmerik et al. 2024).

### Sox2 plasticity in nucleosome binding

Recent insightful work by the Nikolova lab described the first application to PFs, focusing on the nucleosome binding of Sox2 (Malaga Gadea and Nikolova 2023). Motivated by the different observed Sox2 binding sites in the cryo-EM structures described above, they compared Sox2 binding to reconstituted nucleosomes with Sox2 binding motifs at either SHL + 2, (Dodonova et al. 2020), at SHL + 5 or + 6 (Michael et al. 2020). They used a dedicated technique, known as methyl-TROSY (methyl-transverse relaxation optimized spectroscopy) NMR (Tugarinov et al. 2003), to study high-molecular weight systems such as the nucleosome in solution (Kato et al. 2011). This approach requires specific isotope labeling of methyl groups in the protein (in this study those of Ile, Leu, Val, and Ala—“ILVA”), so that only the signals coming from these methyl groups are observable, sculpturing a unique protein fingerprint.

When Sox2-DBD was then titrated with nucleosomes with the binding site at SHL + 5, the ILVA signals in the DNA binding interface showed large chemical shift changes which matched remarkably well with those observed for binding to naked DNA. Interestingly, the signals for V5, which is in the N-terminal tail of the DBD that is ordered in the DNA complex (Reményi et al. 2003), indicated a well-defined and stable complex when using one DNA motif (from the *Fgf4* enhancer), but a less defined and more dynamic complex when using another DNA motif (from the pluripotency-related *Nanog* enhancer). Surprisingly, when the Nanog site was at SHL + 2, the signal of V5 was close to the free state position, indicating that the N-terminal remains disordered and does not significantly contribute to binding. Together, the work by Malaga Gadea et al. highlighted how plasticity in the PF Sox2 translates into flexibility in binding different target sites, at different nucleosomal locations with distinct binding modes and affinities.

### Conformational space of the disordered regions of Sox2

The sensitivity of NMR to intrinsically disordered regions (IDRs) in proteins was exploited in a recent study that integrated NMR, single-molecule Förster resonance energy transfer (smFRET), and coarse-grained MD simulations to offer a new perspective on the role of DNA binding on the exposure of the Sox2 activation domains (Bjarnason et al. 2024). These domains are part of a C-terminal IDR that was shown by NMR and smFRET to transiently collapse on the Sox2 DBD, driven by charge–charge interactions.

Upon binding to nucleosomes or naked DNA, both NMR and smFRET indicated changes in the conformational ensemble of the IDR. Using coarse grain MD simulations, it could be shown that DNA binding causes expansion of the IDR and increased exposure of the second, most C-terminal activation domain (Bjarnason et al. 2024). Therefore, we anticipate that NMR will be instrumental in the future to study PF-mediated nucleosome dynamics considering full-length histone tails and their posttranslational modifications.

## Concluding remarks and outlook

Here we reviewed the current knowledge about the mechanisms by which PFs impact chromatin dynamics. A large gap in knowledge remains between the indirect evidence for the PF-mediated chromatin opening from genome-wide studies and the recent rise in structural studies on PF-nucleosome. The structures, while ground-breaking, present end-states reached after binding and miss out on the flexible components of the systems (histone tails and their posttranslational modifications, the intrinsically disordered regions of PFs). As a result, it is generally hard to rationalize the mechanism of nucleosome distortion or opening. In addition, the dynamical response of the nucleosome may be different in systems using genomic DNA sequences that are targeted in the cell, compared to strong-positioning or optimized sequences.

We anticipate that these limitations can be addressed using an integrated approach combining NMR and MD simulations. Such an approach can yield quantitative structural insights in conformation transitions (Wang et al. 2016; Vallurupalli et al. 2016). Moreover, it can explain how DNA binding alters the conformational ensembles of a PF IDR (Bjarnason et al. 2024). We anticipate that integrated NMR and MD studies can greatly contribute to resolving several open questions:

i) The role of PF plasticity in binding (partial or degenerated) motifs in nucleosomes. So far, the structural data indicate that PFs with two linked DBDs can exploit the flexible linkage between the DBDs to adapt their binding mode from free to nucleosomal DNA. This flexibility may also allow to adjust the binding mode according to the location within the nucleosome. For single-domain DBDs, the adaption may be more localized. For instance, data for Sox2 suggest that structural changes are limited to the N- and C-terminal tails of the DBD. Yet, given that the Sox2 DBD is reportedly highly dynamic, it remains to be established to what extent conformational entropy of the protein is dependent on motif degeneracy or binding site location.
ii) The role of local histone-DNA interface distortions on nucleosome destabilization and opening. So far, it seems as if the structure of the histone core remains largely unaffected by PF binding. However, some PFs distort the DNA and break histone-DNA contacts which in turn may affect the structure of the histone core. Since the strength of the histone-DNA contacts depends on the SHL, it is possible that PF-induced distortions of the histone core are also dependent on the binding location.
iii) The roles of histone tails and PF IDRs in nucleosome binding and opening. Since the histone tails control part of the nucleosome opening and since the tails can be displaced upon PF binding, there is a clear need to determine the changes in conformational space and intra-/intermolecular interactions of the histone tails upon PF binding. Similarly, the IDR regions in the PF could form stabilizing or destabilizing intermolecular interactions with the nucleosome. The IDR regions in *Drosophila* PFs Zelda, Grainy head, and Twist are essential for targeting closed chromatin (Gibson et al. 2024). It is conceivable but not yet addressed that the PF IDRs contribute to chromatin unfolding through excluded volume effects or by interactions with histone tails, other proteins, or DNA.

The next step in bridging the knowledge gap will be to understand how PFs cooperate to open nucleosomes and chromatin fibers. To date, it remains unknown whether DNA binding cooperativity such as that of Oct4 and Sox2 on free DNA plays an important functional role in the recognition of binding sites in the nucleosomes and in nucleosome or chromatin opening. There is no evidence that the cooperativity binding of PFs has a more profound effect on nucleosome structure and dynamics. In fact, the structures of the Oct4-Sox2-nucleosome ternary complexes (Michael et al. 2020) revealed less open nucleosome conformations than those observed in the structures of Oct4-nucleosome (Guan et al. 2023) and Sox2-nucleosome (Dodonova et al. 2020) binary complexes.

Ultimately, it will be essential to study how PF binding modulates the structure of native chromatin fibers. It has been already shown that PFs are able to displace linker histones (Guan et al. 2023) or nucleosomes in non-native fibers (Mivelaz et al. 2020). To study native fibers, coarse-grained MD simulations have been used to simulate entire gene loci (Neguembor et al. 2022) (Portillo-Ledesma et al. 2024). It is possible that such simulations may be combined with solid-state NMR in the future to gain a more accurate view of PF-mediated chromatin dynamics. The last and possibly most difficult step will be to understand how the PF-induced structural changes in nucleosomes and chromatin fibers are linked to chromatin activation and ultimately to gene regulation.

## Data Availability

No new data was generated for this manuscript.
